# The prognostic value of liver function abnormalities in adult secondary hemophagocytic lymphohistiocytosis

**DOI:** 10.3389/fmed.2026.1902118

**Published:** 2026-08-17

**Authors:** Jujuan Wang, Guangli Yin

**Affiliations:** 1 Department of Hematology, The First Affiliated Hospital of Nanjing Medical University, Jiangsu Province Hospital, Nanjing, China; 2 Jiangsu Province Engineering Research Center of Cell and Gene Therapy for hematologic and lymphoid diseases, Nanjing, China; 3 Jiangsu Key Lab of Cancer Biomarkers, Prevention and Treatment, Collaborative Innovation Center for Personalized Cancer Medicine, Nanjing Medical University, Nanjing, Jiangsu, China

**Keywords:** hepatobiliary dysfunction, liver function test abnormalities, lymphoma, mortality, secondary hemophagocytic lymphohistiocytosis

## Abstract

**Background:**

Adult secondary hemophagocytic lymphohistiocytosis (sHLH) is a disorder of immune dysregulation and hyperinflammation that is frequently accompanied by hepatic dysfunction. Evidence describing liver function test (LFT) abnormalities and their prognostic associations in adults with sHLH remains limited.

**Methods:**

In this retrospective study, clinical data and LFT results were obtained at admission from 269 patients with sHLH. Multivariable logistic regression and restricted cubic spline models were used to evaluate the associations between LFTs and in-hospital mortality.

**Results:**

Abnormal alanine aminotransferase (ALT), aspartate aminotransferase (AST), alkaline phosphatase (ALP), and total bilirubin (TBIL) levels were present in 56.5, 75.8, 61.3, and 42.0% of patients, respectively. In multivariable models, higher log-transformed ALP was associated with 28-day mortality (OR 2.84, 95% CI 1.16–7.22; *P* = 0.025), and higher log-transformed TBIL was associated with 7-day mortality (OR 2.77, 95% CI 1.06–7.42; *P* = 0.038) and 28-day mortality (OR 4.84, 95% CI 2.35–10.56; *P* < 0.001). When analyzed as categorical variables, abnormal TBIL was associated with greater odds of 7-day (OR 3.56, 95% CI 1.25–11.27; *P* = 0.022) and 28-day mortality (OR 2.03, 95% CI 1.12–3.71; *P* = 0.020), whereas abnormal ALP was associated with 28-day mortality (OR 2.34, 95% CI 1.26–4.48; *P* = 0.008). Restricted cubic spline analysis showed a non-linear association between ALP and 28-day mortality (*P* for non-linearity = 0.018). Neither ALT nor AST was associated with either outcome after multivariable adjustment.

**Conclusion:**

Abnormal LFTs were common at admission, and ALP and TBIL may be useful prognostic markers of short-term in-hospital mortality in adults with sHLH.

## 1 Introduction

Adult secondary hemophagocytic lymphohistiocytosis (sHLH) is characterized by uncontrolled activation of the lymphoid and mononuclear phagocytic systems, resulting in overwhelming hypercytokinemia and multiple organ failure, including hepatic dysfunction. In 2009, Filipovich suggested that liver involvement should be included in the diagnostic criteria (1). Hepatic injury is common in newly diagnosed sHLH and may present as elevated liver enzymes, hepatomegaly, or progression to acute liver failure (ALF), thereby contributing to high syndrome-related mortality (2). Pathological examination of liver tissue from deceased patients with HLH has revealed substantial hepatic involvement, including necrosis, fibrosis, and cholestasis (3). Our previous study showed that an elevated serum aspartate aminotransferase-to-alanine aminotransferase ratio (AST/ALT) was significantly associated with inferior overall survival (*P* = 0.001) (4). This finding was subsequently supported by Li et al. (5) who reported that all patients with HLH who died within 7 days developed hepatic dysfunction.

Early studies of liver injury in HLH were limited by small sample sizes and analyses of individual markers. Detailed characterization of LFT abnormalities, including ALT, AST, alkaline phosphatase (ALP), and total bilirubin (TBIL), remains limited. More recently, LFTs have been proposed as predictors of liver dysfunction and survival in patients with acute heart failure (6), adult-onset Still's disease (AOSD) (7), and COVID-19 (8). Overall, combined assessment of LFTs, rather than assessment of a single parameter, may facilitate the prompt recognition and early treatment of patients with HLH.

To date, the prevalence and patterns of abnormal LFTs directly associated with in-hospital mortality in sHLH have not been well characterized. Therefore, this study aimed to (1) determine the prevalence of abnormal LFTs at admission; (2) assess the associations of LFTs, analyzed as binary and continuous variables, with in-hospital mortality; and (3) evaluate these associations across prespecified subgroups.

## 2 Patients and methods

### 2.1 Patients and data collection

We retrospectively extracted data from the electronic medical records of consecutive patients with confirmed secondary hemophagocytic lymphohistiocytosis who were treated at the First Affiliated Hospital of Nanjing Medical University from September 2016 to December 2020. The study protocol was approved by the ethics committee of the First Affiliated Hospital of Nanjing Medical University (2019-SR-446). All patients met the HLH-2004 diagnostic criteria (9) and were aged 18 years or older. The exclusion criteria were known hepatic impairment, a history of alcohol abuse, and missing LFT data.

The medical records of all patients were reviewed for demographic characteristics, laboratory findings, and bone marrow aspiration results, as described in our previous studies (4, 10). In addition, tumor or lymph node biopsy findings and PET/CT data were reviewed to confirm lymphoma-associated hemophagocytic lymphohistiocytosis (LHLH).

### 2.2 Definitions

Liver tests included ALT, AST, ALP, and TBIL. Abnormal liver test results were defined according to the laboratory reference ranges as follows: ALT > 50 U/L, AST > 40 U/L, ALP > 120 U/L, and TBIL > 19 μmol/L. For each of the four LFTs, patients were classified as having normal or abnormal values according to the corresponding upper limit of normal. The study outcomes were in-hospital mortality within 7 and 28 days. The time origin was the date of hospital admission. Patients who died in hospital within 7 or 28 days were coded as events for the corresponding binary outcome. Baseline liver function tests were defined as the first measurements obtained within 24 h of hospital admission.

### 2.3 Treatments

Among the 269 patients, 141 (52.4%) received systemic combination chemotherapy as initial therapy for sHLH, 34(12.6%) received HLH-94-based treatment, 30 (11.2%) received glucocorticoids (GC) plus high-dose intravenous immunoglobulin (IVIG), 25 (9.3%) received GC+IVIG + etoposide, 16 (5.9%) received GC + IVIG + etoposide + cyclosporine, 4 (1.5%) patients with progressive multiple organ dysfunction received only high-dose immunoglobulin and glucocorticoid therapy, and 19 (7.1%) patients declined treatment. There were no differences in treatment regimens among the three groups.

### 2.4 Statistical analysis

Categorical variables are presented as percentages and were compared using the chi-squared test. Continuous variables are presented as the mean ± SD or median (IQR), and were compared using Student's *t*-test or the Mann-Whitney *U*-test, as appropriate. The normality of numerical variables was assessed using the Shapiro-Wilk test. Logistic regression was used because the outcomes were binary indicators of in-hospital death within the prespecified 7-day and 28-day windows. Covariates were selected *a priori* based on established prognostic relevance in adult sHLH and previous work. The following confounders were included in the multivariable analyses: age, splenomegaly, log-transformed platelet count, TG, FIB, log-transformed sCD25, log-transformed ferritin, EBV status, etiology and treatment strategies (10). Restricted cubic spline analysis was performed to assess the non-linear relationships between LFTs and the risk of 28-day mortality after adjustment for confounders. Non-linearity was assessed using Wald χ2 tests. Stratified analyses were performed to evaluate the associations between abnormal LFTs and each outcome in the following prespecified subgroups: etiology (LHLH or non-LHLH), sex, age (cut-off: 60 years), ANC (cut-off: 1 × 10^9^/L), FIB (cut-off: 1.5 g/L), TG (cut-off: 3.0 mmol/L), ferritin (cut-off: 5,000 μg/L), sCD25 (cut-off: 36,923 ng/L), and EBV status (no EBV infection or EBV infection).

All statistical analyses were performed using R software, version 3.6.1 (R Foundation for Statistical Computing). A two-sided *P* < 0.05 was considered statistically significant.

## 3 Results

### 3.1 Baseline characteristics stratified by lymphoma association

Of the 269 patients, 171 (63.6%) had LHLH and 98 (36.4%) had non-LHLH (Table 1). Compared with patients with non-LHLH, those with LHLH were more often male (70.2% vs. 45.9%, *P* < 0.001), more frequently required ICU care (21.1% vs. 11.2%, *P* = 0.041), and had higher frequencies of shock (11.7% vs. 4.1%, *P* = 0.035) and splenomegaly (81.9% vs. 53.1%, *P* < 0.001). Patients with LHLH also had higher sCD25 levels and lower platelet counts.

**Table 1:** Baseline demographic, clinical, and laboratory characteristics stratified by lymphoma-associated vs. non-lymphoma-associated sHLH.

Characteristic	Non-lymphoma (*n* = 98)	Lymphoma (*n* = 171)	*P* value
Male, *n* (%)	45 (45.9%)	120 (70.2%)	**< 0.001**
Age, years	52 (39–64)	56 (40–66)	0.560
**Lymphoma stage**, ***n*** **(%)**			**NA**
Stage III	NA	34 (19.9%)	
Stage IV	NA	137 (80.1%)	
**B symptoms**, ***n*** **(%)**	NA	171 (100.0%)	**NA**
ICU requirement, *n* (%)	11 (11.2%)	36 (21.1%)	**0.041**
Sepsis, *n* (%)	5 (5.1%)	17 (9.9%)	0.163
Shock, *n* (%)	4 (4.1%)	20 (11.7%)	**0.035**
**Treatment strategy**, ***n*** **(%)**			**< 0.001**
Chemotherapy/HLH-94	23 (31.1%)	100 (71.9%)	
Dex+IVIg±CsA	40 (54.1%)	32 (23.0%)	
Anti-infection/refuse treatment	11 (14.9%)	7 (5.0%)	
EBV positive, *n* (%)	42 (43.3%)	89 (53.6%)	0.106
Ferritin, ug/L	4,590 (1,501–15,000)	3,035 (1,500–10,083)	0.171
sCD25, ng/L	27,025 (14,506–45,314)	41,166 (25,052–59,678)	**< 0.001**
ANC, × 10^∧^9/L	1.37 (0.75–2.96)	1.07 (0.70–2.12)	0.184
HB, g/L	83 (74–89)	81 (70–88)	0.087
PLT, × 10^∧^9/L	48 (25–80)	37 (21–62)	**0.019**
FIB, g/L	1.28 (0.93–1.94)	1.33 (1.05–2.01)	0.415
TG, mmol/L	2.48 (1.64–3.78)	2.59 (1.78–3.52)	0.907
Splenomegaly, *n* (%)	52 (53.1%)	140 (81.9%)	**< 0.001**
Hemophagocytosis, *n* (%)	77 (78.6%)	124 (72.5%)	0.271

Values are median (IQR) or *n* (%). Percentages are based on non-missing observations. Age was compared with the two-sided Mann-Whitney *U*-test. Categorical variables were compared with Pearson chi-square or Fisher's exact test, as appropriate. Lymphoma stage and B symptoms are not applicable to the non-lymphoma group; *P* values were not calculated. Ferritin, sCD25, ANC, HB, PLT, FIB, and TG are reported as median (IQR). ICU, intensive care unit; EBV, Epstein–Barr virus; HLH-94, hemophagocytic lymphohistiocytosis-94 treatment protocol; Dex, dexamethasone; IVIg, intravenous immunoglobulin; CsA, cyclosporine A; sCD25, soluble CD25/soluble interleukin-2 receptor; ANC, absolute neutrophil count; HB, hemoglobin; PLT, platelet count; FIB, fibrinogen; TG, triglyceride; *n*, number; %, percentage

### 3.2 Prevalence of abnormal LFTs at baseline

**Figure 1:**
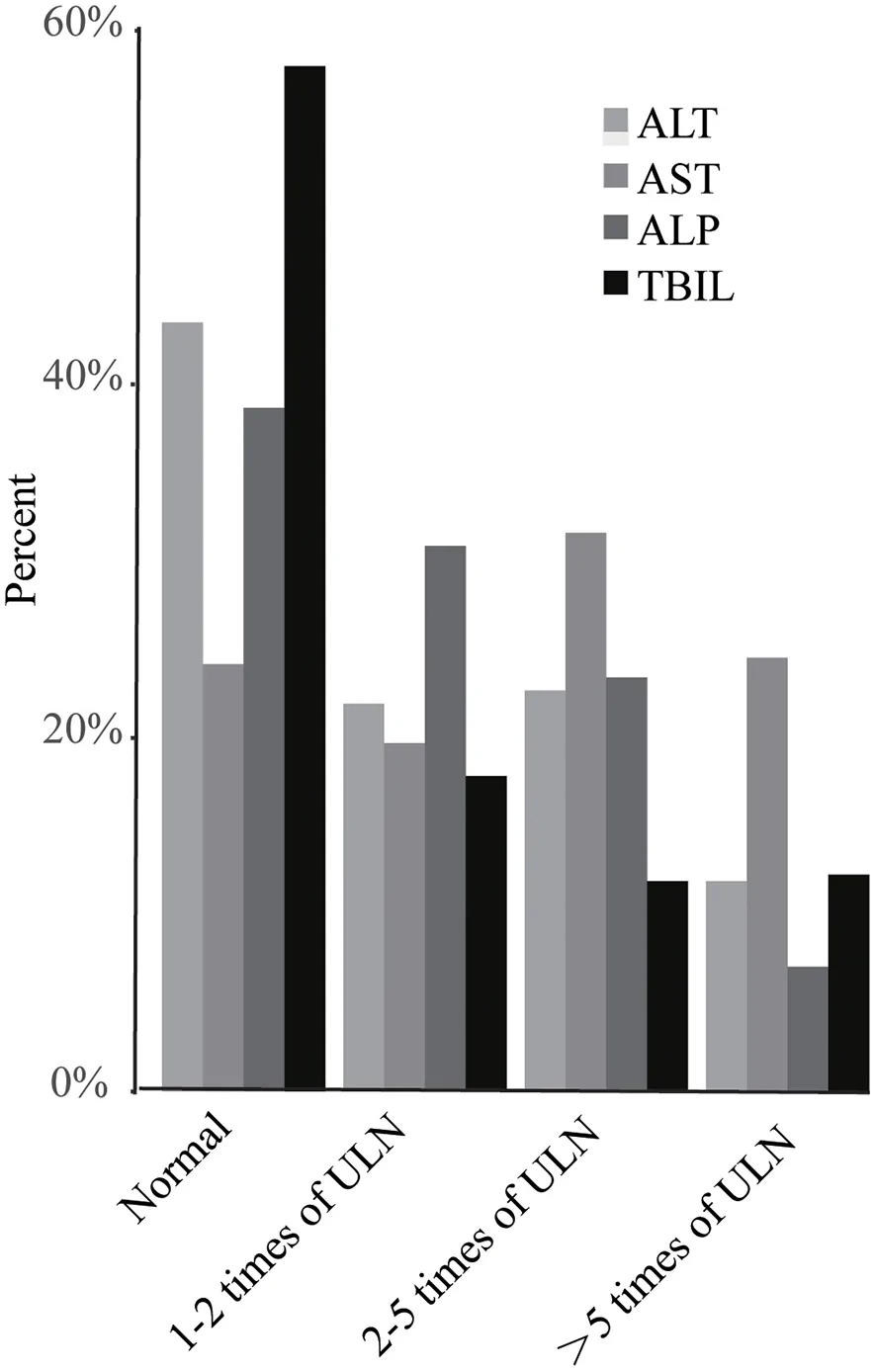
Histogram showing the distributions of baseline liver function tests at admission in patients with sHLH. ALT, alanine aminotransferase; AST, aspartate aminotransferase; ALP, alkaline phosphatase; TBIL, total bilirubin; ULN, upper limit of normal; sHLH, secondary hemophagocytic lymphohistiocytosis.

Among the 269 patients with sHLH enrolled in this study, the median (IQR) baseline values were 61 (21.8–133.2) U/L for ALT, 88.5 (40.5–198.0) U/L for AST, 149.3 (88.0–286.0) U/L for ALP, and 16.5 (10.5–36.9) μmol/L for TBIL. Abnormal liver test results were common at admission: 56.5% had abnormal ALT, 75.8% had abnormal AST, 61.3% had abnormal ALP, and 42.0% had abnormal total bilirubin. Figure 1 shows the distributions of LFT values in the overall cohort.

### 3.3 Clinical characteristics

Table 2 compares baseline characteristics according to each LFT. Patients with abnormal ALT or AST levels were younger, had significantly lower fibrinogen (FIB) levels, and had higher hemoglobin (HB), triglyceride (TG), lactate dehydrogenase (LDH), and ferritin levels than patients with normal ALT or AST levels (all *P* < 0.05). Patients with abnormal ALP had higher TG and LDH levels and lower platelet (PLT) and albumin levels (all *P* < 0.05). Moreover, splenomegaly was more prevalent among patients with abnormal ALP levels than among those with normal ALP levels (77.2% vs. 64.4%, *P* = 0.026). Compared with patients with normal TBIL, those with abnormal TBIL had lower PLT [30.0 (16.0–51.0) vs. 51.0 (31.0–75.0), *P* < 0.001] and higher TG [3.12 (1.93–4.11) vs. 2.36 (1.68–3.23), *P* = 0.002]. Treatment regimens did not differ significantly between patients with abnormal and normal LFTs.

**Table 2:** Baseline demographic, clinical, and laboratory characteristics of sHLH patients by liver function tests (LFTs).

Characteristic	ALT		AST		ALP		TBIL	
	Normal	Abnormal	Normal	Abnormal	Normal	Abnormal	Normal	Abnormal
Male, *n* (%)	71 (60.7)	94 (61.8)	41 (63.1)	124 (60.8)	59 (56.7)	106 (64.2)	89 (57.1)	76 (67.3)
Age, years	61 (48–68)	50 (33–63)^***^	61 (47–67)	52 (35–65)^*^	56 (40–67)	54 (39–65)	54 (38–65)	58 (46–67)
EBV, *n* (%)†	53 (46.9)	78 (52.0)	27 (42.2)	104 (52.3)	48 (48.0)	83 (50.9)	70 (46.4)	61 (54.5)
ANC	1.22 (0.70–2.67)	1.00 (0.72–2.15)	1.07 (0.60–2.50)	1.12 (0.73–2.37)	1.08 (0.68–2.25)	1.14 (0.73–2.38)	1.10 (0.75–2.37)	1.08 (0.62–2.59)
HB	78 (65–85)	84 (76–93)^***^	76 (62–88)	83 (73–89)^**^	83 (74–90)	82 (70–87)	83 (73–89)	82 (69–88)
PLT	43 (21–70)	41 (24–67)	48 (27–76)	39 (23–65)	48 (29–75)	37 (21–68)^*^	51 (31–75)	30 (16–51)^***^
FIB	1.38 (1.08–2.29)	1.25 (0.88–1.75)^**^	1.66 (1.19–2.60)	1.26 (0.93–1.77)^***^	1.39 (1.10–2.10)	1.26 (0.90–1.90)	1.35 (1.09–1.99)	1.27 (0.84–1.92)
TG	2.16 (1.57–3.23)	2.89 (1.92–4.05)^***^	1.89 (1.54–2.74)	2.79 (1.79–3.89)^***^	2.24 (1.64–3.20)	2.87 (1.85–3.91)^**^	2.36 (1.68–3.23)	3.12 (1.93–4.11)^**^
ALB	27.3 (23.3–30.7)	27.5 (24.3–31.1)	27.7 (24.2–31.7)	27.0 (24.2–30.6)	28.5 (24.2–32.9)	26.8 (24.1–30.2)^*^	27.6 (24.2–31.4)	26.9 (24.1–30.8)
LDH	607 (374–929)	810 (479–1711)^***^	389 (283–620)	832 (530–1,627)^***^	623 (376–1,021)	721 (458–1,545)^*^	671 (414–1,136)	704 (414–1,605)
Ferritin	2110 (1207–6267)	5,794 (1,798–15,000)^***^	1,594 (1,044–2,795)	5,068 (1,737–15,000)^***^	2,548 (1,336–12,238)	3,588 (1,637–13,131)	3,114 (1,500–12,278)	4,048 (1,502–13,310)
sCD25	43242 (25812–57509)	41,513 (26,857–49,545)	43,915 (29,674–56,588)	44,069 (27,916-53,160)	33,718 (17,762–52,463)	37,007 (21,358–56,242)	3,4271 (20,589–53,704)	37,470 (17,916–54,492)
Splenomegaly, *n* (%)	86 (73.5)	106 (71.1)	49 (76.6)	143 (70.8)	67 (64.4)	125 (77.2)^*^	111 (71.2)	81 (73.6)
Hemophogcytic, *n* (%)	88 (78.6)	113 (76.4)	51 (79.7)	150 (76.5)	79 (78.2)	122 (76.7)	118 (77.6)	83 (76.9)
Etiology								
LHLH, *n* (%)	80 (68.4)	91 (59.9)	45 (69.2)	126 (61.8)	61 (58.7)	110 (66.7)	93 (59.6)	78 (69.0)
Non-LHLH, *n* (%)	37 (31.6)	61 (40.1)	20 (30.8)	78 (38.2)	43 (41.3)	55 (33.3)	63 (40.4)	35 (31.0)
Chemotherapy, *n* (%)	78 (66.7)	97 (63.8)	45 (69.2)	130 (63.7)	65 (62.5)	110 (66.7)	103 (66.0)	72 (63.7)

ALT, alanine aminotransferase; AST, aspartate aminotransferase; ALP, alkaline phosphatase; TBIL, total bilirubin; EBV, Epstein-Barr virus; ANC, absolute neutrophil count; HB, hemoglobin; PLT, platelet; FIB, fibrinogen; TG, triglyceride; ALB, albumin; LDH, lactate dehydrogenase; sCD25, soluble interleukin−2 receptor; LHLH, lymphoma-associated hemophagocytic lymphohistiocytosis; Non-LHLH, non-lymphoma associated hemophagocytic lymphohistiocytosis. †EBV were available in 263 of 269 patients. Continuous variables represented as mean (IQR). Proportions are expressed as %. ^*^P < 0.05, ^**^P < 0.01, ^***^P < 0.001 when compared with the normal group.

### 3.4 Associations between abnormal liver function tests and in-hospital mortality

**Figure 2:**
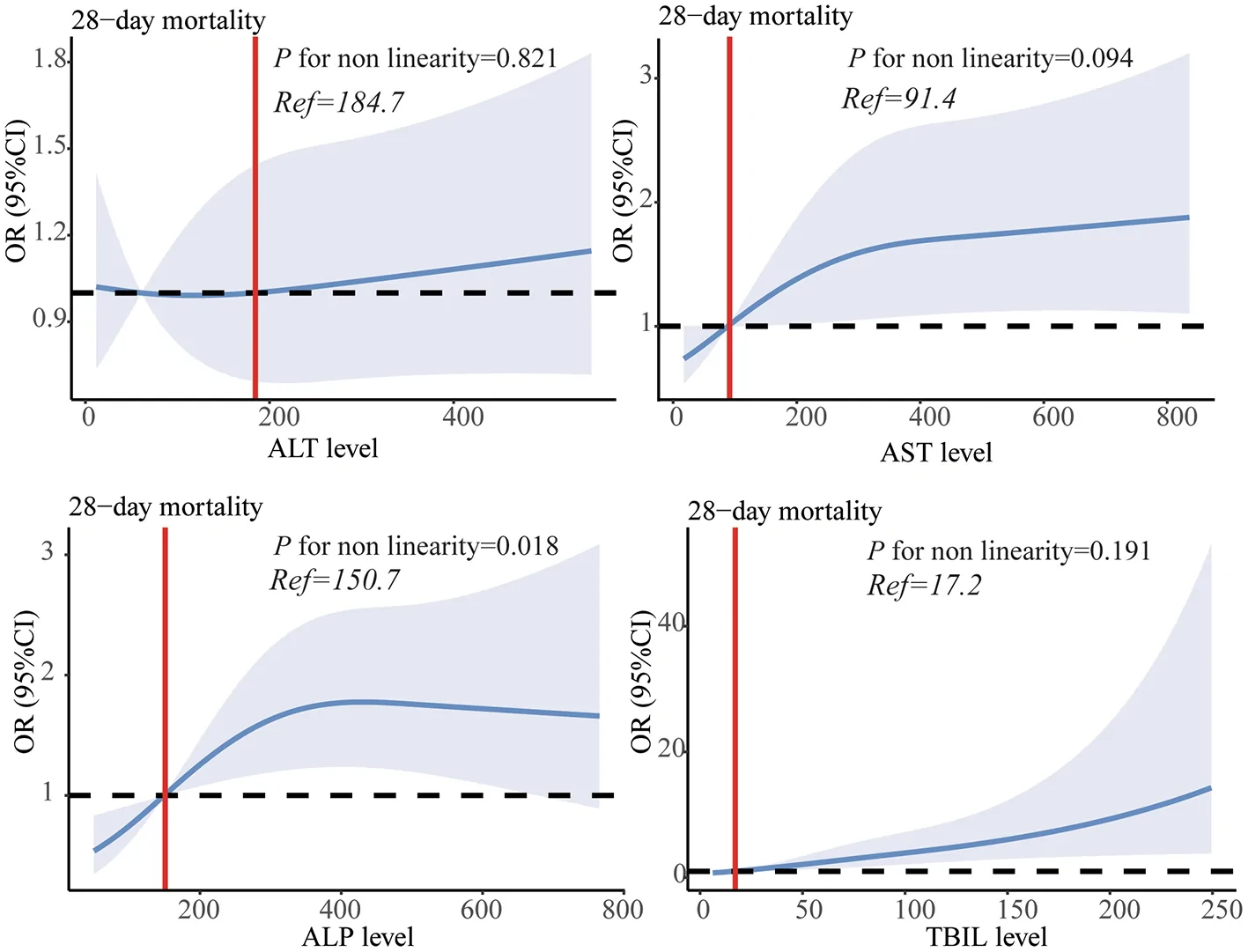
Restricted cubic spline plots of the associations between each LFT parameter and the risk of 28-day mortality in adults with HLH. The blue lines and shaded areas represent the estimated odds ratios and corresponding 95% CIs. OR, odds ratio; CI, confidence interval; ALT, alanine aminotransferase; AST, aspartate aminotransferase; ALP, alkaline phosphatase; TBIL, total bilirubin.

Figure 2 shows restricted cubic spline plots of the odds ratios (ORs) and 95% confidence intervals (CIs) for the associations of each LFT with 28-day mortality. ALP showed a strong non-linear positive association with 28-day mortality (*P* for non-linearity = 0.018). Table 3 presents the unadjusted and multivariable-adjusted analyses of odds ratios (ORs) and 95% confidence intervals (CIs) for the associations between abnormal LFT parameters and the study outcomes. When abnormal LFT parameters were analyzed as continuous variables in multivariable analyses, log-transformed ALT and AST were not associated with either 7 or 28-day in-hospital mortality. In contrast, log-transformed TBIL was associated with 7-day mortality (OR 2.77, 95% CI 1.06–7.42; *P* = 0.038) and 28-day mortality (OR 4.84, 95% CI 2.35–10.56; *P* < 0.001). Log-transformed ALP was associated with 28-day mortality (OR 2.84, 95% CI 1.16–7.22; *P* = 0.025), but not 7-day mortality. When analyzed as categorical variables, abnormal ALT and AST were not associated with either outcome. Abnormal TBIL was associated with 7-day mortality (OR 3.56, 95% CI 1.25–11.27; *P* = 0.022) and 28-day mortality (OR 2.03, 95% CI 1.12–3.71; *P* = 0.020). Abnormal ALP was associated with 28-day mortality (OR 2.34, 95% CI 1.26–4.48; *P* = 0.008), but not 7-day mortality.

**Table 3:** Clinical outcomes for sHLH patients with abnormal liver function tests (LFT > ULN).

Variable	Unadjusted			Adjusted		
	OR	95%CI	*P*-value	OR	95%CI	*P-*value
7-day mortality†						
Log of ALT	1.02	0.47–2.25	0.96	1.07	0.36–3.22	0.905
Log of AST	3.14	1.41–7.0	**0.01**	2.70	0.88–8.72	0.086
Log of ALP	0.93	0.32–2.74	0.89	0.70	0.16–2.92	0.631
Log of TBIL	2.73	1.31–5.70	**0.01**	2.77	1.06–7.42	**0.038**
28–day mortality‡						
Log of ALT	0.97	0.57–1.66	0.93	1.27	0.65–2.55	0.485
Log of AST	1.68	0.96–2.95	0.07	1.40	0.65–3.06	0.393
Log of ALP	2.61	1.23–5.55	**0.01**	2.84	1.16–7.22	**0.025**
Log of TBIL	5.38	2.85–10.17	**< 0.001**	4.84	2.35–10.56	**< 0.001**
7-day mortality†						
Abnormal ALT	0.99	0.47–2.08	0.98	0.72	0.25–2.04	0.532
Abnormal AST	2.43	0.82–7.20	0.11	1.50	0.39–7.76	0.585
Abnormal ALP	1.45	0.66–3.19	0.36	1.89	0.67–5.86	0.245
Abnormal TBIL	2.58	1.20–5.53	**0.01**	3.56	1.25–11.27	**0.022**
28-day mortality‡						
Abnormal ALT	1.01	0.61–1.65	0.98	1.08	0.58–2.02	0.803
Abnormal AST	1.64	0.90–2.97	0.10	1.40	0.68–2.96	0.370
Abnormal ALP	1.96	1.17–3.30	**0.01**	2.34	1.26–4.48	**0.008**
Abnormal TBIL	2.53	1.53–4.19	**< 0.001**	2.03	1.12–3.71	**0.020**

sHLH, secondary hemophagocytic lymphohistiocytosis; OR, Odds ratio; CI, Confidence interval; ALT, alanine aminotransferase; AST, aspartate aminotransferase; ALP, alkaline phosphatase; TBIL, total bilirubin; LFT, liver function tests. †7-day mortality adjusted for: age, splenomegaly, log PLT, FIB, log Ferritin, EBV status, etiology, treatment strategies. ‡28-day mortality adjusted for: age, splenomegaly, log PLT, TG, FIB, logsCD25, log Ferritin, EBV status, etiology, treatment strategies. Abnormal ALT and AST values considered greater than 50 U/L and 40 U/L; Abnormal ALP and TBIL considered greater than 120 U/L and 19 umol/L.

### 3.5 Stratified associations between abnormal liver function tests and outcomes

Further stratified analyses according to sHLH etiology are shown in Table 4. No clear associations between abnormal LFTs and 7-day or 28-day mortality were observed in either LHLH or non-LHLH patients, except for abnormal TBIL. After adjustment for other prognostic variables, the risk associated with abnormal TBIL for 7-day mortality was significantly higher in the non-LHLH group [23.58 (3.24–530.68)] than in the LHLH group [0.89 (0.25–3.17); *P* for interaction = 0.029].

**Table 4:** Multivariate analysis by etiologies of sHLH and binary liver function test (LFT) (interaction *P*: etiologies × LFT).

Variable	LHLH			OR	Non-LHLH	*P*	*P* for interaction
	OR	95% CI	*P*		95% CI		
7-day mortality†							
Abnormal ALT	1.46	0.39–5.85	0.580	0.43	0.09–1.82	0.252	0.499
Abnormal AST	2.08	0.36–20.22	0.461	2.54	0.40–49.77	0.403	0.886
Abnormal ALP	1.23	0.34–5.01	0.763	1.85	0.46–9.33	0.408	0.417
Abnormal TBIL	0.89	0.25–3.17	0.859	23.58	3.24–530.68	**0.009**	**0.029**
28-day mortality‡							
Abnormal ALT	1.35	0.61–2.98	0.457	0.41	0.13–1.25	0.124	0.417
Abnormal AST	1.61	0.66–4.11	0.304	0.89	0.24–3.40	0.857	0.776
Abnormal ALP	1.70	0.77–3.88	0.196	3.11	1.03–10.46	0.051	0.238
Abnormal TBIL	1.21	0.57–2.58	0.615	2.95	1.03–8.74	0.046	0.078

sHLH, secondary hemophagocytic lymphohistiocytosis; LHLH, lymphoma-association hemophagocytic lymphohistiocytosis; Non-LHLH, non-lymphoma association hemophagocytic lymphohistiocytosis; OR, Odds ratio; CI, Confidence interval; ALT, alanine aminotransferase; AST, aspartate aminotransferase; ALP, alkaline phosphatase; TBIL, total bilirubin.

^†^7-day mortality adjusted for: age, splenomegaly, log PLT, FIB, log Ferritin, EBV status, etiology, treatment strategies.

^‡^28-day mortality adjusted for: age, splenomegaly, log PLT, TG, FIB, logsCD25, log Ferritin, EBV status, etiology, treatment strategies.

**Figure 3:**
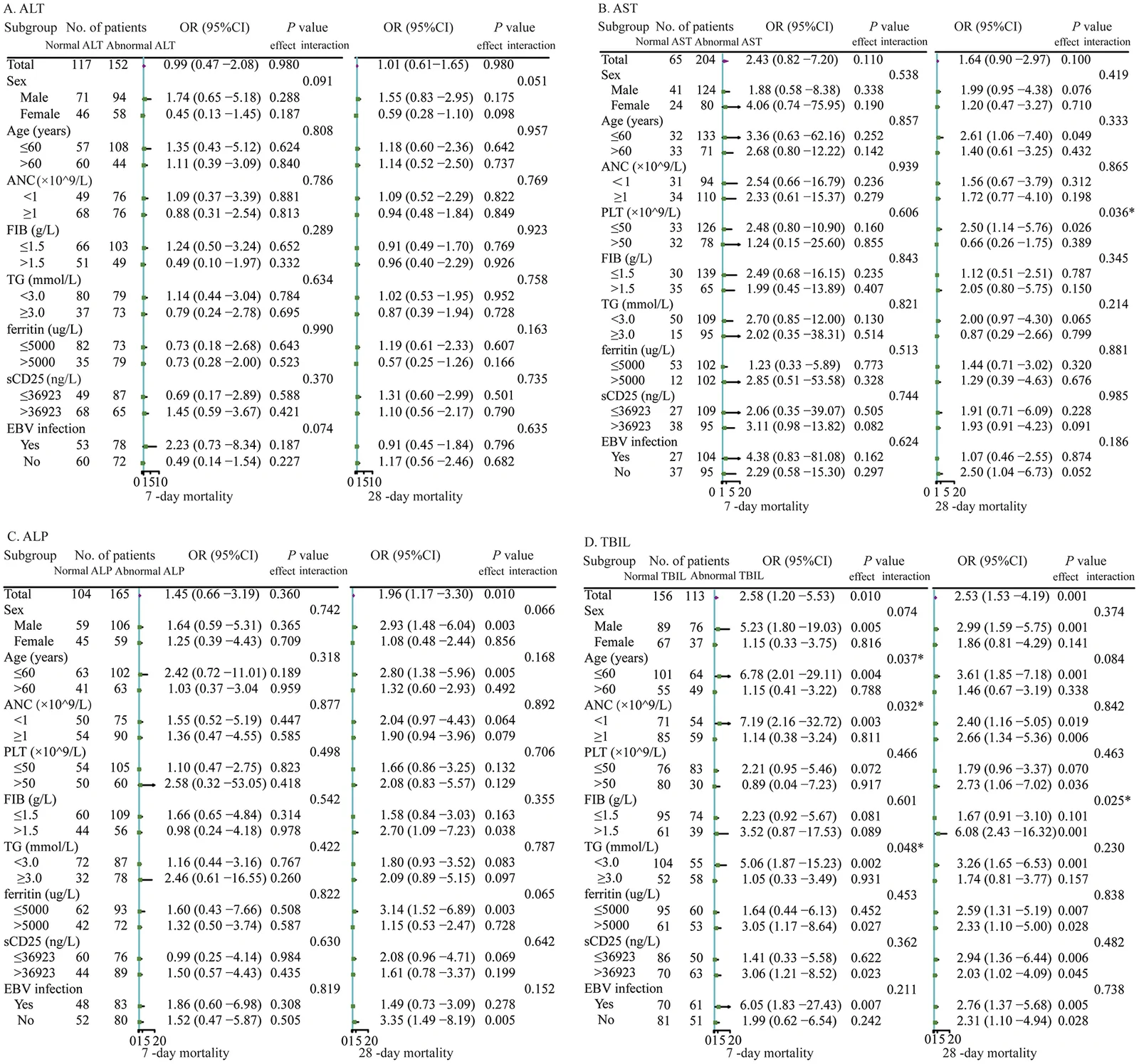
Associations of abnormal vs. normal LFTs at admission with 7-day and 28-day mortality. **(A)** ALT; **(B)** AST; **(C)** ALP; **(D)** TBIL, OR, odds ratio; CI, confidence interval; ALT, alanine aminotransferase; AST, aspartate aminotransferase; ALP, alkaline phosphatase; TBIL, total bilirubin; ANC, absolute neutrophil count; FIB, fibrinogen; TG, triglyceride; sCD25, soluble interleukin-2 receptor; EBV, Epstein-Barr virus.

Figure 3 presents unadjusted subgroup associations between abnormal vs. normal LFTs and 7 and 28-day mortality. In the subgroup with platelet counts ≤ 50 × 10^∧^9/L, abnormal AST was associated with 28-day mortality, with evidence of interaction by platelet count (*P* for interaction = 0.036, Figure 3B). For TBIL, interactions were observed with age ≤ 60 years, ANC < 1 × 10^∧^9/L, and TG < 3 mmol/L for 7-day mortality and with fibrinogen > 1.5 g/L for 28-day mortality (*P* for interaction = 0.037, 0.032, 0.046, and 0.025, respectively; Figure 3D). No significant interactions were observed for ALT or ALP (Figure 3A and C).

## 4 Discussion

This analysis of LFTs in sHLH yielded three major findings: (i) abnormal LFTs were common at admission; (ii) abnormal TBIL was independently associated with 7-day and 28-day mortality, whereas abnormal ALP was independently associated with 28-day mortality and showed a strong positive L-shaped association with 28-day mortality; no such associations were observed for abnormal ALT or AST; and (iii) subgroup analyses generally showed a consistent trend toward a greater risk of in-hospital mortality associated with abnormal LFTs across prespecified patient groups. Overall, abnormal ALP and TBIL appeared to identify patients with sHLH at high risk of in-hospital mortality. ALP is not specific for cholestasis and may also increase with systemic inflammation. Its association with mortality may therefore partly reflect the overall inflammatory burden of sHLH rather than isolated hepatobiliary dysfunction. Elevated TBIL may occur relatively late in severe sHLH, after substantial hepatocellular injury and/or cholestatic dysfunction has developed, and may therefore indicate advanced systemic disease. The observed association with mortality should consequently be interpreted as prognostic rather than causal.

We observed a relatively high prevalence of abnormal LFTs in patients with sHLH at admission (56.5, 75.8, 61.3, and 42.0% with abnormal ALT, AST, ALP, and TBIL, respectively). Notably, elevated AST was more common than elevated ALT, which may reflect severe hepatocellular injury, including mitochondrial damage. The rate of TBIL abnormalities in our study was similar to that previously reported (11). However, elevated ALP was included in our definition of abnormal LFTs, whereas it was not assessed in most previous studies. Much of the previous evidence linking LFT abnormalities to poor prognosis has been based on individual LFT parameters. In this study, we assessed a hepatobiliary dysfunction (HBD) profile comprising ALT, AST, ALP, and TBIL. Previous research has suggested a 4-fold increase in the risk of death among patients with sHLH and HBD (3). Therefore, close monitoring of patients with abnormal liver test results at admission is warranted.

The mortality rate was significantly higher in children with ALF-HLH (7/8, 88%) than in children with HLH without ALF (4/15, 27%, *P* = 0.009) (12). In a previous sHLH study, the estimated short-term probability of survival was 70.1% for 30-day mortality rate and 61% for 7-day mortality rate (5). In line with these findings, the 28-day mortality rate in our cohort was 60.6%. We observed that higher ALP and TBIL values, analyzed as continuous variables, were associated with a higher risk of 28-day mortality (PALP = 0.025; PTBIL < 0.001) after adjustment for preexisting prognostic factors. The 7-day mortality rate was 88.1% in our cohort. Higher TBIL levels were strongly associated with 7-day mortality. Similar results were observed when these markers were analyzed as categorical variables. Clinically, abnormal LFTs have been widely used to assess liver injury and predict survival outcomes in various diseases. Biegus et al. showed that abnormal LFTs at baseline were associated with a higher risk of in-hospital death in patients with acute heart failure (6). Piano et al. similarly showed that patients with COVID-19 and abnormal LFTs had a higher risk of transfer to the ICU and death (13). AOSD is a potential cause of sHLH, and patients with higher LFT values have a poorer prognosis (7). In our study, patients with abnormal LFTs, particularly elevated ALP and TBIL, had a worse prognosis. We therefore suggest that LFT abnormalities should receive greater consideration when assessing in-hospital mortality risk in sHLH, as liver involvement is already included in the diagnostic criteria for HLH (14). In addition, multiple analyses and RCS modeling showed that LFTs were associated with 7 and 28-day mortality. A non-linear relationship was observed between ALP and 28-day mortality. Subgroup analyses were performed according to LHLH status, an important etiologic factor in sHLH (15). Abnormal TBIL was more strongly associated with 7-day mortality in non-LHLH patients than in LHLH patients (OR = 23.58, *P* for interaction = 0.029). Furthermore, after stratification by clinical characteristics, abnormal LFTs were associated with in-hospital mortality compared with normal LFTs.

The increased risk of mortality associated with abnormal LFTs might be attributable to unchecked antigen presentation, hypercytokinemia, dysregulated cytotoxicity mediated by natural killer (NK) cells and CD8^+^T cells, and disruption of intrinsic hepatic metabolic pathways (2, 16). Histologic assessment may assist in the evaluation of patients with sHLH and abnormal LFTs, but liver biopsy is often not feasible because of severe coagulopathy and platelet dysfunction at admission (2). Common histological findings reported by Chen et al. included portal and sinusoidal infiltration by CD3^+^, CD8^+^, and granzyme cytotoxic lymphocytes, together with CD68^+^ hemophagocytic histiocytes (17). Some studies suggest that hepatic dysfunction may be driven by both immune effector cells and non-immune hepatic cells, including hepatocytes, liver sinusoidal endothelial cells (LSECs), and hepatic stellate cells (HSCs) (2).

Our study provides a detailed description of LFT profiles in sHLH in a relatively large sample. We found that abnormal ALP and TBIL were independently associated with short-term mortality in sHLH. However, our study has several limitations. We lacked data on histologic parameters (e.g., inflammatory cell infiltrate counts and liver parenchymal necrosis) and cytokine profiles and therefore could not further investigate their relationships with LFTs. Liver dysfunction in sHLH is likely multifactorial and may reflect systemic hyperinflammation, endothelial injury, infectious or autoimmune triggers, and prior or concurrent treatment-related effects. Because liver biopsy was generally not feasible in patients with severe thrombocytopenia and coagulopathy, the relative contribution of these mechanisms could not be determined. In addition, residual measured and unmeasured confounding could not be completely excluded because of the retrospective design, may affect both mortality and liver dysfunction in patients with sHLH, although multivariable models were used to adjust for potential confounders, including etiology, lymphoma stage/group, ICU requirement, sepsis, and shock. Finally, this was an observational study, and the observed associations should not be interpreted as causal. Well-designed, multicenter, prospective studies are needed to validate our findings.

## 5 Conclusion

Elevated ALP and TBIL at admission were independently associated with short-term in-hospital mortality in adults with sHLH. Abnormal admission LFTs may serve as prognostic markers, and clinicians should remain alert to the potential for rapid clinical deterioration.

## Data Availability

The raw data supporting the conclusions of this article will be made available by the authors, without undue reservation.
